# Supplementary material for: Reproducibility of dynamic contrast enhanced MRI derived transfer coefficient Ktrans in lung cancer
Source: PLoS One. 2022 Mar 8;17(3):e0265056. doi: 10.1371/journal.pone.0265056 (PMC8903254; doi:10.1371/journal.pone.0265056)
Supplement: Attachment — Submitted filename: Rebuttal letter.docx [file pone.0265056.s002.docx]

**Rebuttal Letter to manuscript PONE-D-21-31257:**

“Reproducibility of dynamic contrast enhanced MRI derived transfer coefficient K^trans^ in lung cancer”

Cologne, 08 February 2022

Dear Prof. Baltzer,

We are pleased that our manuscript is being considered for publication in PLOS ONE. We would like to thank the reviewer for the helpful and well-founded suggestions for improving our manuscript. In the following, all requirements of the journal and the reviewer will be commented on in note form. All content-related changes have been highlighted in the revised manuscript with track changes. We hope to meet all your requirements and would be honoured to continue to be considered for publication in PLOS ONE.

Kind regards,

Jean-Philip Weber

**Responses to Review Comments**

1. **The abstract conveys the most important information in a very comprehensible manner. There is a minor grammatical mistake in the last sentence, as it says „DCE MRI offers with Ktrans a reliable quantitative biomarker“, which should rather read „With Ktrans, DCE MRI offers a reliable quantitative biomarker“, i.e. the words should be repositioned.**

*We apologize for the grammatical mistake. The sentence structure has been modified in the revised manuscript according to your suggestion. The correction was made both in the abstract on page 2, line 41 and in the discussion on page 16, line 340.*

2. **The authors should look into the distributions of Ktrans values around their means per timepoint, specifically their deviations from normality, since the calculation of confidence intervals for ICCs assumes a normal distribution. Since Ktrans values are zero-bound, a truly normal distribution is not possible. For Fig. 3, a graphical representation of medians (i.e. a box plot in the style of Tukey) should be chosen, rather than means and SDs.**

*Thank you for the legitimate suggestion for improvement. In both Table 2 and Fig 3, due to the heterogenous distribution, the means and standard deviations for K^trans^ were misleading. Table 2 now shows the median values of K^trans^ and the interquartile ranges (IQR). Fig 3 has also been renewed and now shows the K^trans^ values in form of boxplots in the style of Tukey.*

3. **The dataset is quite heterogenous, as it consists of both extra- and intrapulmonary lesions with variable sizes. Due to the low number of intrapulmonary lesions, reliability measures on these might not be conclusive. The authors might consider to exclude these lesions from their analyses. Alternatively, for table 3 and 4, lesions could be grouped according to both size and location, i.e. showing data for intrapulmonary lesions larger than 3 cm, extrapulmonary lesions larger than 3 cm, intrapulmonary lesions smaller than 3 cm and extrapulmonary lesions smaller than 3 cm.**

*We thank you for the concrete and legitimate proposals. The division of the lesions and the resulting structure of our work was subject of intense debates in our research group. In fact, the extrapulmonary metastases are small in number, which reduces statistical significance. This point is therefore explicitly taken up again in the discussion section on page 14, line 302-309. Nevertheless, we did not want to undermine the poor reproducibility of extrapulmonary metastases, with the aim of pointing out that future measurements of such lesions may require adjustments to protocols and study settings.*

*Furthermore, we also considered subdividing the extrapulmonary lesions according to their size. In contrast to the intrapulmonary lesions, however, there does not seem to be a meaningful cut-off analogous to the T stage or the RECIST criteria. This would make any subdivision look arbitrary. Since in our dataset the lymph nodes are already rather small (2.6 ± 0.5 cm) and the bone metastases are rather larger (4.3 ± 0.8 cm), a natural split is already given here in a certain respect.*

*While we fully understand your concerns, we hope this provides some explanation of our perspective to illustrate why we want to keep the extrapulmonary lesion as part of our work and refrain from making any further subdivisions.*

4. **Table 4 is titled „Intraclass agreement“, whereas the corresponding paragraph in the text is titled „Intrarater agreement“. The authors should change the title of the table to avoid confusion.**

*Thank you for the kind notice. To avoid possible confusion, it is important to us to use a uniform vocabulary. The designation was therefore adjusted according to your suggestion.*

5. **In the discussion, the authors claim that Ktrans is „probably the most used and most established DCE-MRI biomarker“, to justify why they chose this parameter over other semiquantitative biomarkers. Is this claim based on purely experience, or if there literature available?**

*We acknowledge that this was a vague wording. References 28 (Di N et al.) and 29 (Jiang T et al.) have been added to support our statement. The corresponding changes were added to the reference list.*

6. **This might not concern the authors directly, but I was not forwarded the supporting information and can therefore not validate that all data is fully available. For this reason I chose „No“ in the corresponding review box.**

*According to the PLOS Data Policy the full dataset of K^trans^ values underlying the statistical ICC and wCOV calculation has been added to the Supporting information named “S1_Dataset”. The dataset contains K^trans^ values measured by every single reader of all 107 tumor lesions sorted by location and size.*

**Adjustments according to Journal Requirements**

1. **PLOS ONE's style requirements**

*The title page has been adjusted according to the PLOS ONE style template. Authors are now given with their full names. An affiliation was added to author Judith Eva Spiro.on page 1, line 13.*

2. **Reference list review**

The reference list was checked for retractions and completeness. The references 28 and 29 have been added according to the reviewer requirements on page 21. Furthermore, the bibliography style has been adjusted according to the PLOS ONE style requirements.

3. **PLOS Data Policy requirements**

The full dataset underlying the calculations of ICCs and wCOV has been added to Supporting Information under the name “S1_Dataset” as requested by the PLOS Data Policy
